# Growth and chemotaxis of nematodes reduced upon exposure to Third Fork Creek surface water

**DOI:** 10.1186/s12302-021-00579-8

**Published:** 2022-01-14

**Authors:** Carresse Gerald, Boris Deshazo, Hayden Patterson, Porché Spence

**Affiliations:** 1 Environmental, Earth and Geospatial Sciences, North Carolina Central University, 1801 Fayetteville Street, 2205 Mary Townes Science Complex, Durham, NC 27707 USA; 2 Science and Research Summer Program, NSF-CREST, North Carolina Central University, Durham, NC 27707 USA; 3 Biological and Biomedical Sciences, North Carolina Central University, Durham, NC 27707 USA

**Keywords:** Nematode, Third Fork Creek, Biological model, Ecotoxicology, Freshwater

## Abstract

**Background:**

Third Fork Creek is a historically impaired urban stream that flows through the city of Durham, North Carolina. *Caenorhabditis elegans* (*C. elegans*) are non-parasitic, soil and aquatic dwelling nematodes that have been used frequently as a biological and ecotoxicity model. We hypothesize that exposure to Third Fork Creek surface water will inhibit the growth and chemotaxis of *C. elegans*. Using our ring assay model, nematodes were enticed to cross the water samples to reach a bacterial food source which allowed observation of chemotaxis. The total number of nematodes found in the bacterial food source and the middle of the plate with the water source was recorded for 3 days.

**Results:**

Our findings suggest a reduction in chemotaxis and growth on day three in nematodes exposed to Third Fork Creek water samples when compared to the control (*p* value < 0.05). These exploratory data provide meaningful insight to the quality of Third Fork Creek located near a Historically Black University.

**Conclusions:**

Further studies are necessary to elucidate the concentrations of the water contaminants and implications for human health. The relevance of this study lies within the model *C*. *elegans* that has been used in a plethora of human diseases and exposure research but can be utilized as an environmental indicator of water quality impairment.

## Background

*Caenorhabditis elegans* are a useful nematode model for genotoxicity, molecular biology [23], and neurological disorders such as Alzheimer’s and Parkinson’s disease [16, 24]. *C.* *elegans* are effective eukaryotic models because a large portion of their genome is evolutionary conserved and 83% of their proteome is homologous to humans [22]. *C. elegans* also can be found naturally in soil and water and have been identified in leaf litter and gastropods [4, 18, 32]. These nematodes have been utilized in several environmental toxicology studies to evaluate toxicity of soils [2, 15, 20, 21], sediments [19, 34, 35] and water [17, 27, 36]. *C. elegans* assist in maintaining soil health by regulating bacteria populations and by indirectly supporting biodiversity. Studies concluded *C. elegans* are a representative model for ecotoxicity [3, 14]. In the study conducted by Hitchcock et al., nematodes were exposed to several composite water samples from five points from within the wastewater treatment plant system. Using a 72-h nematode mortality test, nematodes experiences increased mortality when exposed to wastewater entering the wastewater treatment plant. In the study conducted by Mutwakil et al., transgenic nematodes were exposed to five water samples collected from the River Carnon in England, which is known to have ancient mining history. Transgenic expression was observed in nematodes exposed to all five samples, with the least amount of expression found in nematodes exposed to water samples containing less contaminants. *C. elegans* are a prodigious model due to the ease of culture in laboratory settings, can be maintained at 25 °C, consume bacteria, and have a short well-studied lifespan [23].

In this study, *C. elegans* are used to investigate toxicity of a historically impaired urbanized watershed in Durham, North Carolina. The number of sampling locations throughout the TFC watershed has declined from 7 locations during the even monitoring years [8, 33] to one consistent location during the odd monitoring years [11]. In 2019, the City of Durham tested one location within the TFC watershed and reported a water quality index value of 76 due to poor bacteria levels, fair nutrient and turbidity levels, and poor aquatic life [11]. The significant reduction in sampling locations within the TFC watershed is concerning, because TFC flows into Jordan Lake, which serves as a drinking water source for several communities located in Wake, Durham and Orange counties [13]. Since TFC runs adjacent to North Carolina Central University (NCCU), a sampling site located near the campus was selected for this exploratory study. We hypothesized the water collected from this site along TFC would inhibit *C. elegans* chemotaxis and growth. For this study, a ring assay was utilized to measure the chemotactic and growth behavior of the *C. elegans* exposed to TFC surface water samples.

## Methods

### Study area

**Fig. 1:**
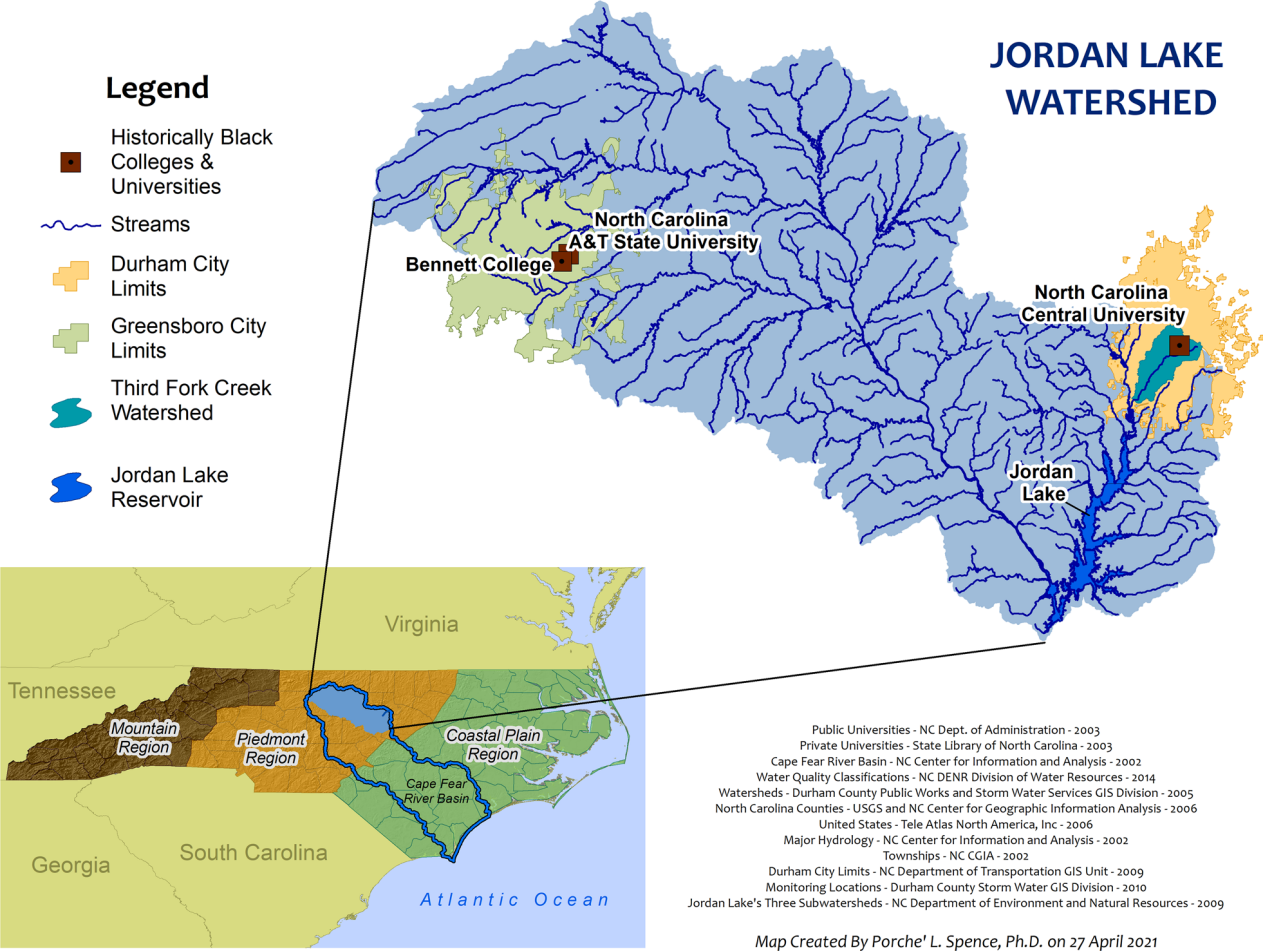
Jordan Lake Watershed including Third Fork Creek

Third Fork Creek (TFC) watershed is located in the Cape Fear River Basin and drains 16.6 square miles of the city of Durham, North Carolina, before reaching Jordan Lake and eventually the Atlantic Ocean (Fig. 1). The predominate land use is a combination residential, commercial and vacant unmanaged space [10]. TFC watershed is classified as protected upstream (WS-V) and nutrient sensitive waters (NSW) [10, 28] TFC drains into a drinking water source (Jordan Lake) that is protected for wading, boating, fishing, wildlife, fish consumption and requires additional nutrient management to reduce the excessive growth of aquatic vegetation [13].

TFC has a history of suffering from a variety of pollution sources (Table 1). Multiple sites are monitored during the even years within the TFC watershed by collecting and analyzing stream samples for biochemical oxygen demand, nutrients (nitrogen/phosphorus), bacteria (fecal coliform), clarity (turbidity), and metals. The presence of aquatic life, such as aquatic insects or benthic macroinvertebrates, is used as water quality indicators. Individually these water quality parameters are used to evaluate the health of the streams and determine if they are meeting the standards for their intended uses. Collectively these parameters are used to assign a Water Quality Index (WQI), which is “one tool that takes different ways to measure water quality and combines them into a single score” that can range between less than 60 (fail) to 100 (excellent) [8]. Between 2010 and 2020 (even year water quality monitoring), the mean WQI value for the TFC watershed has been 72, which can be interpreted as a grade “C” for water quality [6].

**Table 1:** Water quality index (WQI) values, number of sampling locations, pollution sources, and parameter rating for Third Fork Creek Watershed between 2010 and 2020

Watershed	Year	Average WQI	Number of sampling locations	Pollution sources	Bacteria	Nutrients	Turbidity	Aquatic Life	References
Third Fork Creek	2010	71	7	Cooking grease and food oil Erosion and sedimentation Improper disposal of yard waste illicit mobile car washing Private sewer overflow Public sanitary sewer over flow Sanitary sewer line breaks	Poor	–	Good	Poor	City of Durham Stormwater and GIS Services [38]
Third Fork Creek	2012	73	7	Cooking grease and food oil Erosion and sedimentation Improper disposal of yard waste Illicit mobile car washing Private sewer overflow Public sanitary sewer over flow Sanitary sewer line breaks	Poor	–	Good	–	City of Durham Stormwater and GIS Services [6]
Third Fork Creek	2014	71	–	–	–	–	–	–	City of Durham Stormwater and GIS Services [8]
Third Fork Creek	2016	76	7	Cooking grease Erosion and sedimentation Illicit mobile car washing Improper disposal of yard waste Paint spills Petroleum spills Private sewer overflow Public sanitary sewer over flow Sanitary sewer line breaks	Poor	Fair	Fair	Poor	City of Durham Stormwater and GIS Services [8]
Third Fork Creek	2018	66	7	Cooking grease Erosion and sedimentation Petroleum spills Private sewer overflow Public sanitary sewer over flow	Poor	Fair	Fair	Poor	City of Durham Stormwater and GIS Services [10]
Third Fork Creek	2020	75	7		Poor	Fair	Good	Poor	City of Durham Stormwater and GIS Services [13]

#### Water collection and sampling

**Fig. 2:**
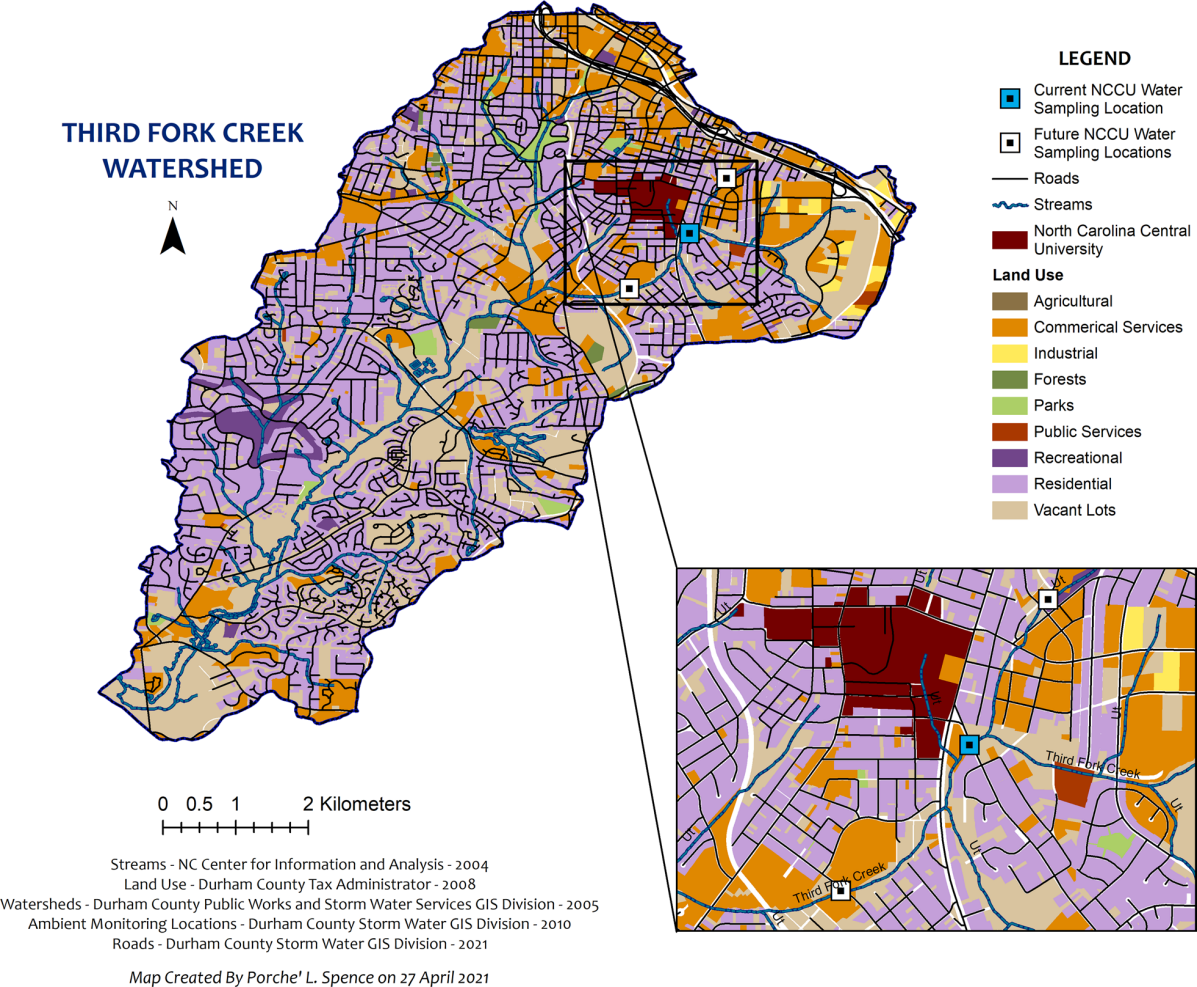
Third Fork Creek land use and NCCU current and potential sampling locations

Grab water samples were collected from one sampling location along TFC (Fig. 2) during the summer of 2019 on June 24 and July 8 and during the winter of 2020 on February 17, February 24, March 2, and March 9. Plastic 50-ml conical tubes were used to collect surface water samples from TFC. Samples were immediately placed on ice in a cooler for travel to the laboratory and stored at 4 ˚C. The winter 2020 sampling period was halted after March 9, 2020 due to the COVID-19 pandemic. The summer 2019 water sampling was conducted as a feasibility study. The water sampling was resumed in winter 2020 with the intention to collect weekly samples and subsequent exposures until the end of July 2020 (summer). The sampling in 2020 was proposed to examine differences between seasons.

**Fig. 3:**
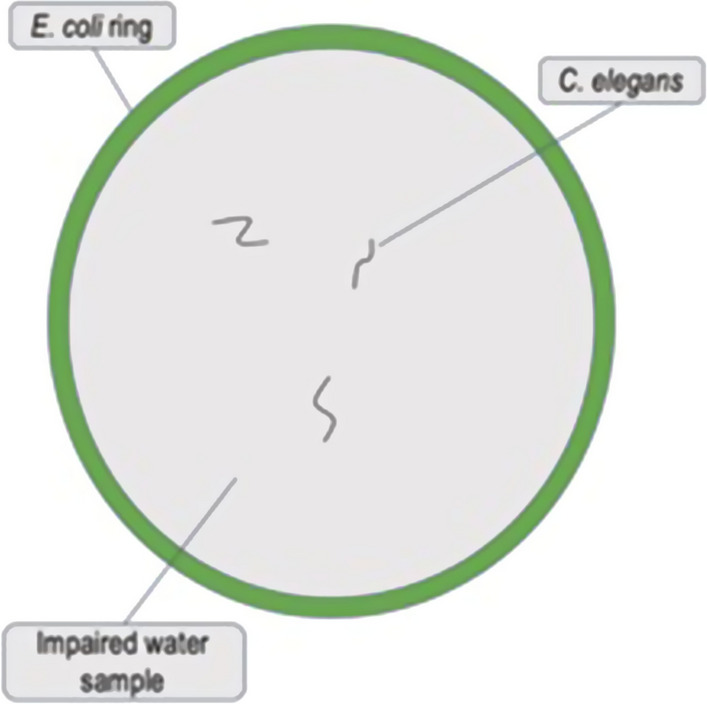
Ring assay plate

#### *C. elegans* inoculation and culture

*C. elegans* (N2 strain), *E. coli* (K-12 strain) and Nematode Growth Agar (NGA) were purchased from Carolina Biological Supply Company (Burlington, NC, USA). *E. coli* was aseptically inoculated onto NGA plates and incubated overnight at 37 °C to create bacteria lawn plates to support and maintain nematodes until time of experiments. Nematodes were inoculated onto bacteria lawn plates and maintained at 25 °C.

#### Three-day nematode growth assay

A ring assay model (Fig. 3, [39]) was used to analyze nematode chemotaxis and growth. Chemotaxis is the movement of motile cells or organisms in response to a chemical stimulus. Growth is defined as the reproduction of the nematodes. An NGA plate was inoculated with *E. coli* around the ring of the plate to entice nematodes to cross the NGA plate (Control), sterile water or TFC sample. The plate was incubated overnight at 37 °C. The following day, a 100 µl volume of sterile water or TFC water sample (water sample was refrigerated immediately after sample collection and used within 24 h for nematode exposure) was added to the middle of the plate. Immediately following the addition of sterile water or TFC water sample, nematodes were inoculated with a sterile toothpick from nematode bacteria lawn growth plates onto the designated ring assay plates. A range of 20–30 nematodes were seeded on each plate. Nematodes in the middle, ring and total number of live nematodes were counted each day post-exposure for 3 days. Chemotaxis shown in the results as nematodes present in the *E. coli* ring and growth was shown as total amount of live nematodes on the entire plate.

### Statistical analysis

The assays were conducted in triplicate in independent times for reproducibility assessment. Each ring assay plate was inoculated with nematodes. Three ring assay plates were used for the control group which consist of no water or TFC sample. Sterile water was used on another three ring assay plates. Three ring assay plates were used for the treatment group which consisted of the TFC water sample.

The Control (no water in the middle of the Ring Assay plates), sterile water (sterile water in the middle of the Ring Assay plates) and TFC water sample (collected surface water from TFC in the middle of the Ring Assay plates) were assessed each week following grab water sample collection. A one-way ANOVA and Tukey’s multiple comparisons post hoc test was employed via GraphPad Prism version 9 to analyze nematode chemotaxis (nematodes present in the *E. coli* ring) and growth (total number of live nematodes on the entire plate) (Fig. 3) [39].

## Results

**Fig. 4:**
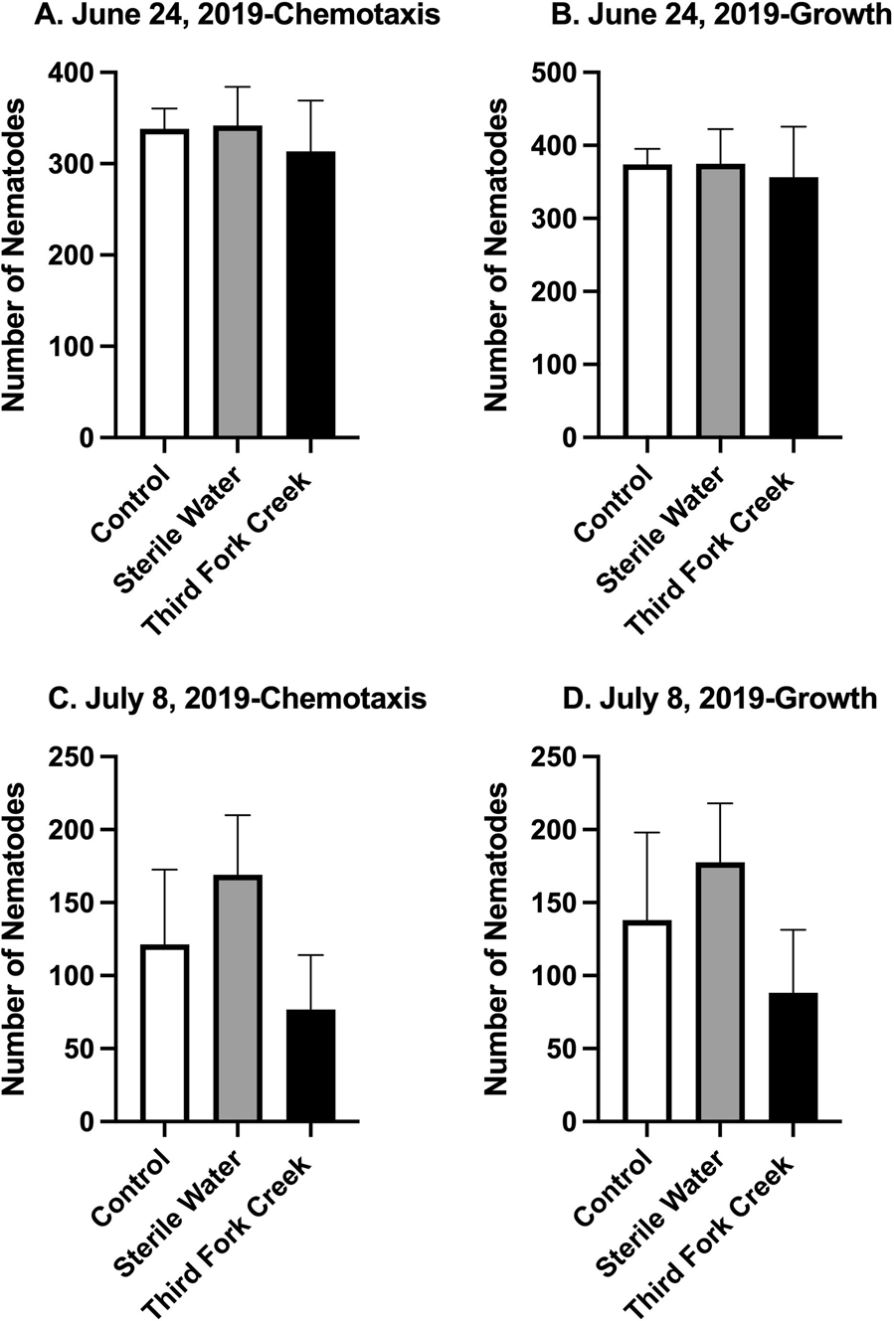
No effect of TFC water samples on nematode chemotaxis and growth for summer sample collections. **A** *C. elegans* were exposed to surface water collected from Third Fork Creek on June 24, 2019 for 3 days. To determine chemotaxis, nematodes present in the bacterial ring on each of the 3 days was recorded and the total number of nematodes in the bacterial ring on day three is presented in **A**. No significant difference was found when comparing Control and Sterile water, Control and Third Fork Creek water sample and Sterile Water and Third Fork Creek water sample (*p* > *0.05*). **B** *C. elegans* were exposed to surface water collected from Third Fork Creek on June 24, 2019 for 3 days. To determine growth, live nematodes on each plate were counted and recorded for each of the 3 days and the total number of live nematodes on day three is presented in **B**. No significant difference was found when comparing Control and Sterile water, Control and Third Fork Creek water sample and Sterile Water and Third Fork Creek water sample (*p* > *0.05*). **C** *C. elegans* were exposed to surface water collected from Third Fork Creek on July 8, 2019 for 3 days. To determine chemotaxis, nematodes present in the bacterial ring on each of the 3 days was recorded and the total number of nematodes in the bacterial ring on day three is presented in **C**. No significant difference was found when comparing Control and Sterile water, Control and Third Fork Creek water sample and Sterile Water and Third Fork Creek water sample (*p* > *0.05*). **D** *C. elegans* were exposed to surface water collected from Third Fork Creek on July 8, 2019 for 3 days. To determine growth, live nematodes on each plate were counted and recorded for each of the 3 days and the total number of live nematodes on day three is presented in **D**. No significant difference was found when comparing Control and Sterile water, Control and Third Fork Creek water sample and Sterile Water and Third Fork Creek water sample (*p* > *0.05*). Significance was determined under a one-way ANOVA and Tukey’s post hoc test. Data are presented as the mean ± SEM for **A**–D, *n* = 3

The data from summer 2019 (June 24 and July 8, 2019) water sampling, collection and 3-day exposure are presented in Fig. 4. To determine chemotaxis, nematodes present in the bacterial ring on each of the 3 days was recorded and the total number of nematodes in the bacterial ring on day three is presented in Fig. 4A. No significant difference was found when comparing Control and Sterile water, Control and Third Fork Creek water sample and Sterile Water and Third Fork Creek water sample (*p* > *0.05*) (Table 2). In Fig. 4B the growth of nematodes were analyzed. Live nematodes on each plate were counted and recorded for each of the 3 days and the total number of live nematodes on day three is presented in Fig. 4B. No significant difference was found when comparing Control and Sterile water, Control and Third Fork Creek water sample and Sterile Water and Third Fork Creek water sample (*p* > *0.05*), data shown in Table 2. In Fig. 4C, *C. elegans* exposed to Third Fork Creek water samples (July 8, 2019) for 3 days. Although there appears to be a decrease in nematodes in the bacterial ring, the data were not significantly different when comparing; Control and Third Fork Creek water sample and Sterile Water and Third Fork Creek water sample (*p* > *0.05*); data shown in Table 2. Similarly, nematodes exposed to Third Fork Creek water samples show a trend of decreasing growth of nematodes (total live nematodes), however no significant differences were found when comparing Control and Sterile water, Control and Third Fork Creek water sample and Sterile Water and Third Fork Creek water sample (*p* > *0.05*); data shown in Table 2. Generally, nematodes in the control and sterile water treatment groups are expected to reach the bacterial ring as well as reproduce over the 3-day life cycle. Overall nematode chemotaxis and growth were not affected by the TFC exposure.

**Table 2:** Comparison of nematode chemotaxis and growth 72 h post-exposure to Third Fork Creek water samples collected in June and July 2019

Date	Behavior parameters	Tukey’s multiple comparison’s test	Mean difference	*p* value
24-June-2019	Chemotaxis	Day 3-control vs. sterile water	− 3.667	0.9979
		Day 3-control vs. Third Fork Creek (TFC)	24.67	0.9125
		Day 3-sterile water vs. TFC	28.33	0.8867
24-June-2019	Growth	Day 3-control vs. sterile water	− 0.6667	< 0.9999
		Day 3-control vs. TFC	17.67	0.9664
		Day 3-sterile water vs. TFC	18.33	0.9639
8-July-2019	Chemotaxis	Day 3-control vs. sterile water	− 47.67	0.7317
		Day 3-control vs. TFC	44.67	0.7588
		Day 3-sterile water vs. TFC	92.33	0.3563
8-July-2019	Growth	Day 3-control vs. sterile water	− 39.67	0.8370
		Day 3-control vs. TFC	49.67	0.7601
		Day 3-sterile water vs. TFC	89.33	0.4458

**Fig. 5:**
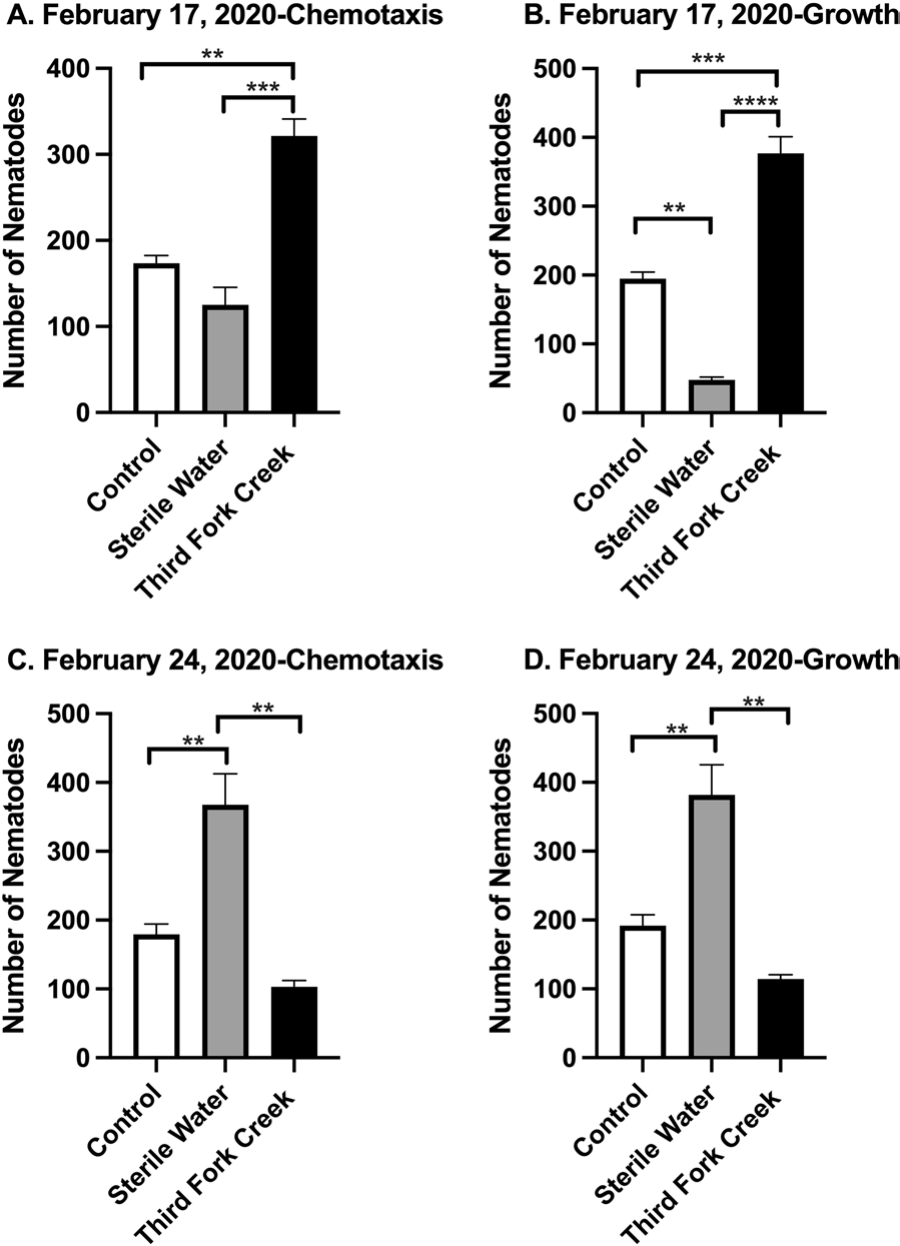
Nematode chemotaxis and growth decreased post-exposure to TFC Surface Water Collected in February 2020. *C. elegans* were exposed to surface water collected from Third Fork Creek on February 17 (**A, B**) and February 24 (**C, D**), 2020 for 3 days. **A** To determine chemotaxis, nematodes present in the bacterial ring on each of the 3 days was recorded and the total number of nematodes in the bacterial ring on day three is presented in **A**. No significant differences were found when comparing Control and Sterile water (*p* = 0.2025). Significant differences were found between Control and Third Fork Creek water sample (** denotes *p* = 0.0023) and Sterile Water and Third Fork Creek water sample (*** denotes *p* = 0.0005). **B** To determine growth, live nematodes on each plate were counted and recorded for each of the 3 days and the total number of live nematodes on day three is presented in **B**. Significant differences was found when comparing Control and Sterile water (** denotes *p* = 0.0013), Control and Third Fork Creek water sample (*** denotes *p* = 0.0004) and Sterile Water and Third Fork Creek water sample (**** denotes *p* < *0.0001*). **C** To determine chemotaxis, nematodes present in the bacterial ring on each of the 3 days was recorded and the total number of nematodes in the bacterial ring on day three is presented in **C**. No significant difference found when comparing Control and Third Fork Creek water sample and Sterile Water. Significant differences were found when comparing Control and Sterile water (** denotes *p* = *0.0074*) and Sterile Water and Third Fork Creek water sample (** denotes *p* = *0.0013*). **D** To determine growth, live nematodes on each plate were counted and recorded for each of the 3 days and the total number of live nematodes on day three is presented in **D**. No significant difference was found when comparing Control and Third Fork Creek water sample. Significant differences between Control and Sterile water (** denotes *p* = *0.0060*), Sterile Water and Third Fork Creek water sample (** denotes *p* = *0.0010*). Significance was determined under a one-way ANOVA and Tukey’s post hoc test. Data are presented as the mean ± SEM for **A**–**D**, *n* = 3

Nematodes exposed to TFC surface water collected in February 2020 are shown in Fig. 5. *C. elegans* were exposed to surface water collected from TFC on February 17, 2020 (Fig. 5A, B) and February 24, 2020 (Fig. 5C, D) for 3 days. Nematodes present in the bacterial ring were recorded and the total number in the bacterial ring on day three was used to measure chemotaxis. When comparing the Control and Sterile water groups, no significant differences between the means were found (*p* = 0.2025, Table 3). However, there was a significant increase of nematodes exposed to the TFC water sample compared to the control (*p* = 0.0023, Table 3) and Sterile Water groups (*p* = 0.0005, Table 3). The growth of nematodes was analyzed via counting all live nematodes on each plate for the 3 days. The total number of live nematodes on day three is shown in Fig. 5B. There was a significant decrease in nematodes exposed to sterile water compared to control (*p* = 0.0013, data shown in Table 3) and the TFC water sample (*p* = 0.0004, Table 3). A significant increase of nematodes was also observed in the TFC water sample when compared to the control (*p* = 0.0004, Fig. 5C). There was not a significant difference when comparing Control and TFC water sample (*p* = 0.2116, Table 3). Significant differences were found when comparing Control and Sterile water (*p* = *0.0074*) and Sterile Water and TFC water sample (*p* = *0.0013*). In Fig. 5D**,** there was not a significant difference for the nematode growth between the Control and TFC water sample collected in February 24, 2020 (*p* = 0.1852, Table 2). Significant increases in nematode growth was observed in the Sterile water treatment group when compared to the Control (*p* = *0.0060*) and TFC water sample (*p* = *0.0010*).

**Table 3:** Comparison of nematode chemotaxis and growth 72 h post-exposure to Third Fork Creek water samples collected in February and March 2020

Date	Behavior parameters	Tukey’s multiple comparison’s test	Mean difference	*p* value
17-February-2020	Chemotaxis	Day 3-control vs. sterile water	48.33	0.2025
		Day 3-control vs. Third Fork Creek (TFC)	− 148.0	0.0023
		Day 3-sterile water vs. TFC	− 196.3	0.0005
17-February-2020	Growth	Day 3-control vs. Sterile Water	147.0	0.0060
		Day 3-control vs. Third Fork Creek (TFC)	− 182.0	0.0004
		Day 3-sterile water vs. TFC	− 329.0	< 0.0001
24-February-2020	Chemotaxis	Day 3-control vs. sterile water	− 188.3	0.0074
		Day 3-control vs. Third Fork Creek (TFC)	76.0	0.2116
		Day 3-sterile water vs. TFC	264.3	0.0013
24-February-2020	Growth	Day 3-control vs. sterile water	− 190.0	0.0060
		Day 3-control vs. Third Fork Creek (TFC)	77.67	0.1852
		Day 3-sterile water vs. TFC	267.7	0.0010
	Chemotaxis	Day 3-control vs. sterile water	− 141.0	0.0086
2- March-2020		Day 3-control vs. Third Fork Creek (TFC)	75.33	0.1065
		Day 3-sterile water vs. TFC	216.3	0.0010
2-March-2020	Growth	Day 3-control vs. sterile water	− 137.7	0.0207
		Day 3-control vs. Third Fork Creek (TFC)	77.33	0.1614
		Day 3-sterile water vs. TFC	215.0	0.0024
9-March-2020	Chemotaxis	Day 3-control vs. sterile water	− 140.3	0.0002
		Day 3-control vs. Third Fork Creek (TFC)	69.33	0.0089
		Day 3-sterile water vs. TFC	209.0	< 0.0001
9-March-2020	Growth	Day 3-control vs. Sterile Water	− 133.0	0.0009
		Day 3-control vs. Third Fork Creek (TFC)	79.33	0.0125
		Day 3-sterile water vs. TFC	212.0	< 0.0001

**Fig. 6:**
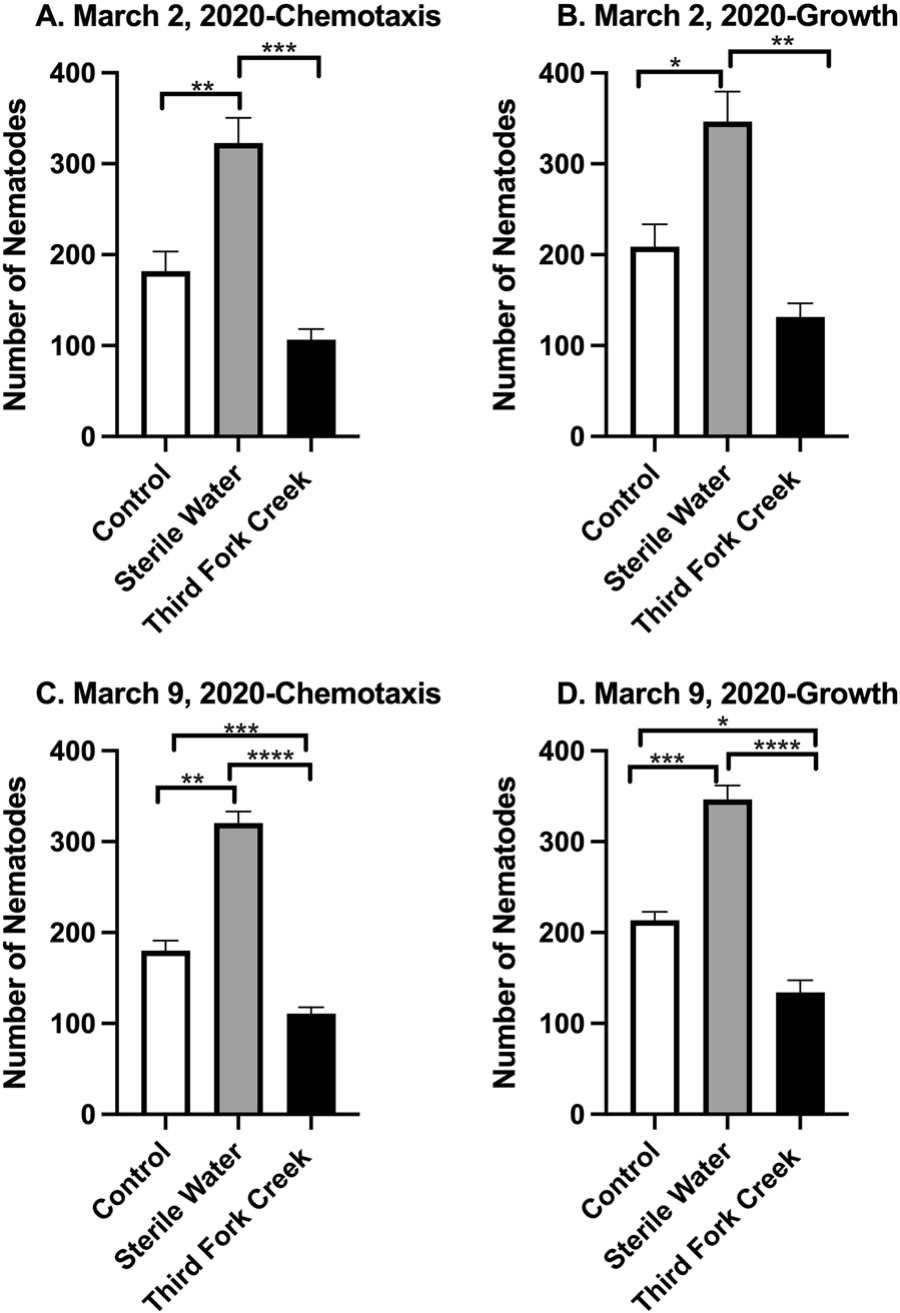
Nematode chemotaxis and growth decreased post-exposure to Third Fork Creek Surface Water collected in March 2020. *C. elegans* were exposed to surface water collected from Third Fork Creek on March 2 (**A, B**) and 9 (**C, D**), 2020 for 3 days. **A** To determine chemotaxis, nematodes present in the bacterial ring on each of the 3 days was recorded and the total number of nematodes in the bacterial ring on day three is presented in **A**. Significant differences were found when comparing Control and Sterile water (** denotes *p* = 0.0086) and Sterile Water and Third Fork Creek water sample (*** denotes *p* = 0.0010). No significant difference was found between Control and Third Fork Creek water sample (*p* = 0.1065). **B** To determine growth, live nematodes on each plate were counted and recorded for each of the 3 days and the total number of live nematodes on day three is presented in **B**. Significant differences were found when comparing Control and Sterile water (* denotes *p* = 0.0207) and Sterile Water and Third Fork Creek water sample (**p denotes *p* = *0.0024*). No significant differences were found between Control and Third Fork Creek water sample (*p* = 0.1614). **C** To determine chemotaxis, nematodes present in the bacterial ring on each of the 3 days was recorded and the total number of nematodes in the bacterial ring on day three is presented in **C**. Significant differences were found when comparing Control and Third Fork Creek water sample and Sterile Water (*** denotes *p* = 0.0089), Control and Sterile water (** denotes *p* = *0.0002*) and Sterile water and Third Fork Creek water sample (**** denotes *p* < *0.0001*). **D** To determine growth, live nematodes on each plate were counted and recorded for each of the 3 days and the total number of live nematodes on day three is presented in **D.** Significant differences were found when comparing Control and Sterile water sample (*** denotes *p* = 0.0009), Control and Third Fork Creek water sample (* denotes *p* = 0.0125) and Sterile Water and Third Fork Creek water sample (**** denotes *p* < *0.0001*). Significance was determined under a one-way ANOVA and Tukey’s post hoc test. Data are presented as the mean ± SEM for **A**–**D**, *n* = 3

*C. elegans* were exposed to surface water collected from Third Fork Creek on March 2, 2020 (Fig. 6A, B) and March 9, 2020 ( Fig. 6C, D) for 3 days. To examine chemotaxis, nematodes present in the bacterial ring on each of the 3 days was recorded and the total number of nematodes in the bacterial ring on day three is presented in Fig. 6A. No significant difference was found between Control and TFC water sample (*p* = 0.1065, Table 3). Nonetheless, nematodes exposed to sterile water experienced higher numbers of nematodes in the bacterial ring compared to Control (*p* = 0.0086, Table 3). A significant decrease in nematodes exposed to TFC water sample (compared to sterile water), *p* = 0.0010 was also noted. Growth of nematodes (total number of live nematodes on day 3) was recorded and is presented in Fig. 6B. Significant increases of nematodes were found in the Sterile Water treatment group compared to the Control group (*p* = 0.0207) and TFC water sample (*p* = *0.0024*). No significant differences were found between Control and TFC water sample (*p* = 0.1614). In Fig. 6C, nematodes present in the bacterial ring on each of the 3 days were recorded and the total number of nematodes in the bacterial ring on day three is presented in Fig. 6C. Significant increases of Sterile Water compared to Control (*p* = 0.0002) and TFC water sample (*p* < 0.0001). TFC exposed nematodes also experienced a decrease in numbers when compared to the Control nematodes (*p* = 0.0089, Table 3). The growth of nematodes exposed to TFC water collected March 9, 2020 is documented in Fig. 6D. TFC water significantly reduced the growth of nematodes when comparing to Control (*p* = 0.0125) and Sterile water (*p* < 0.0001). Also, an increase of nematodes exposed to sterile water, compared to control were observed (*p* = 0.0009).

## Discussion

This exploratory study utilized the free-living nematode, *C. elegans*, which represents the largest animal phylum on earth [38], to study the impact of freshwater exposure on chemotaxis and growth. Nematodes are categorized as meiofauna and meiobenthos which are ecologically important and known sentinels of pollution [10]. The February and March 2020 data (Figs. 5C, D, 6A–D) showed TFC exposure led to a significant decrease of nematode chemotaxis and growth. Third Fork Creek is known as a historically impaired, urbanized watershed therefore these data can provide insight to how meiofauna are affected by freshwater samples with a history of pollution (Table 1). Chemotaxis impairment of meiofauna can be imperative to microecosystems as they are often the intermediate food source between bacteria and macrofauna [38]. Chemotaxis is a foraging mechanism utilized by *C. elegans* to locate food sources often bacteria. The ring assay in this model demonstrates the ability for *C. elegans* to chemotax across TFC samples and locate *E. coli*. As shown in Figs. 5 and 6, the number of nematodes reaching the *E. coli* dropped significantly. These findings represent the real-world impact of impaired water on nematode communities. Disturbing the nematode populations, could be detrimental to a freshwater ecosystem as many larger fauna prey on meiofauna and therefore the absence can cause disruptions to the microcosm as well as the stream’s global ecosystem. TFC has a history of suffering from poor aquatic life (Table 1). This strengthens our findings which shows TFC decreasing chemotaxis and growth. Poor aquatic life is indicative of poor water quality and ecological health [8].

In addition to the pollutants listed by the City of Durham Stormwater and GIS Services in the State of the Stream reports (2011–2021), pharmaceuticals such as caffeine, antibiotics, fire retardants and pesticides were also found in the TFC watershed [31]. *C. elegans* exposed to caffeine experience food aversion behavior [26] and decreased larval development [25]. Nematodes feed off bacteria commonly found in the soil and water and antibiotics could decrease the levels of this food source. Also, certain pesticides are broad spectrum and can have nematicidal effects which could explain the decrease of nematode chemotaxis and growth.

Another interesting finding in this study, was the ability of the nematodes to thrive in the sterile water control when compared to the control (Figs. 5C, D, 6A–D). It is known that *C. elegans* can locomote on land or aqueous solutions, but has been documented to locomote faster in water than land [37]. These results explain the increase in chemotaxis and growth due to nematodes swimming faster in the water and also validate the TFC water samples collected on February 24, March 2 and March 9 (Figs. 5C, D, 6A–D) impeding chemotaxis and growth. Even though, the mean values of sterile water compared to the control and TFC were not significantly different, a similar trend was observed for the sample collected on July 8, 2019 (Fig. 4C, D).

Chemotaxis and growth were not impacted by the sample collected on June 24, 2019 (Fig. 4A, B). The environmental conditions such as precipitation, temperature could have influenced the parameters we measured. A similar unexpected result was observed from samples collected on February 17, 2020 (Fig. 5A, B). Nematodes exposed to TFC water collected on February 17, 2020 had an increase of nematodes reaching the food source (chemotaxis) as well as an increase of total live nematodes (growth) on day 3, which shows nematodes were capable of reproducing in that environment. It is thought that the TFC samples could have been diluted from previous precipitation events.

Unfortunately, the COVID-19 pandemic paused the water collection and nematode analysis in winter 2020. We anticipate expanding to four collection sites and commencing weekly collections during the 2022 year. This study will be expanded to elucidate genetic and proteomic differences in nematodes exposed to grab water samples from TFC watershed. In addition, to the nematode analysis, we will also investigate common water quality parameters such as pH, dissolved oxygen and analysis of common nutrients and pollutants. The water samples collected in this study are not of a pure sample. We do not know the internal concentrations of the constituents in collected samples. The volume used in the ring assay is very small in comparison to the whole sample collected. Since this is an exploratory study, we used 100% of the sample. For future observations we will seek to establish ECx values regarding the nematode experiments.

Increasing the number of sampling locations, frequency of sample collection and using *C. elegans* as an environmental indicator species will assist in evaluating seasonal differences observed from our current TFC dataset. Therefore, future sampling locations will be selected upstream and downstream of the current NCCU water sampling location (Fig. 2) to focus on traditionally underserved minority communities near North Carolina Central University, a Historically Black University. Future nematode observations will include counting eggs to better understand the growth and analyzing locomotion to observe chemotaxis more efficiently. Evidence provided by this study will supplement the sampling efforts conducted by the city of Durham and can be used to educate residents about potential health implications.

## Conclusions

The sampling locations in this preliminary study are limited, but the information provided in this article supports the need for including additional sites along TFC in future studies. Our data show *C. elegans* can be a useful model to analyze the impact of freshwater samples on nematode chemotaxis and growth. Even though this study is focused on a local stream, our methods can be used to study water quality impairment in freshwater systems at larger scales and in different locations using a very inexpensive model organism.

## Data Availability

All data generated or analyzed during this study are included in this published article.
